# Analysis of laboratory testing results collected in an enhanced chlamydia surveillance system in Australia, 2008–2010

**DOI:** 10.1186/1471-2334-14-325

**Published:** 2014-06-12

**Authors:** Wayne Dimech, Megan SC Lim, Caroline Van Gemert, Rebecca Guy, Douglas Boyle, Basil Donovan, Margaret Hellard

**Affiliations:** 1 NRL, 4th Floor Healy Building, 41 Victoria Parade, Fitzroy 3065, Australia; 2 Burnet Institute, Centre for Population Health, 85 Commercial Road, Melbourne, Victoria 3004, Australia; 3 Monash University, School of Population Health and Preventive Medicine, 99 Commercial Rd, Melbourne 3004, Australia; 4 The Kirby Institute, Sexual Health Program, University of New South Wales, Sydney, NSW 2052, Australia; 5 GRHANITE Health Informatics Unit, Rural Health Academic Centre, Melbourne Medical School, University of Melbourne, 49 Graham Street, Shepparton, Victoria 3630, Australia; 6 Sydney Sexual Health Centre, Sydney Hospital, Sydney, NSW 2000, Australia

**Keywords:** *Chlamydia trachomatis*, ACCESS laboratory network, Surveillance, Sexually transmitted infection

## Abstract

**Background:**

Chlamydial infection is the most common notifiable disease in Australia, Europe and the US. Australian notifications of chlamydia rose four-fold from 20,274 cases in 2002 to 80,846 cases in 2011; the majority of cases were among young people aged less than 29 years. Along with test positivity rates, an understanding of the number of tests performed and the demographics of individuals being tested are key epidemiological indicators. The ACCESS Laboratory Network was established in 2008 to address this issue.

**Methods:**

The ACCESS Laboratory Network collected chlamydia testing data from 15 laboratories around Australia over a three-year period using data extraction software. All chlamydia testing data from participating laboratories were extracted from the laboratory information system; patient identifiers converted to a unique, non-reversible code and de-identified data sent to a single database. Analysis of data by anatomical site included all specimens, but in age and sex specific analysis, only one testing episode was counted.

**Results:**

From 2008 to 2010 a total of 628,295 chlamydia tests were referred to the 15 laboratories. Of the 592,626 individual episodes presenting for testing, 70% were from female and 30% from male patients. In female patients, chlamydia positivity rate was 6.4% overall; the highest rate in 14 year olds (14.3%). In male patients, the chlamydia positivity rate was 9.4% overall; the highest in 19 year olds (16.5%). The most common sample type was urine (57%). In 3.2% of testing episodes, multiple anatomical sites were sampled. Urethral swabs gave the highest positivity rate for all anatomical sites in both female (7.7%) and male patients (14%), followed by urine (7.6% and 9.4%, respectively) and eye (6.3% and 7.9%, respectively).

**Conclusions:**

The ACCESS Laboratory Network data are unique in both number and scope and are representative of chlamydia testing in both general practice and high-risk clinics. The findings from these data highlight much lower levels of testing in young people aged 20 years or less; in particular female patients aged less than 16 years, despite being the group with the highest positivity rate. Strategies are needed to increase the uptake of testing in this high-risk group.

## Background

*Chlamydia trachomatis* is the most commonly notified infection in Australia [1] with notifications increasing from 20,274 cases in 2002 to 80,846 cases in 2011 [1]. The World Health Organization estimates there are over 100 million new cases globally each year [2,3] resulting in a substantial cost to health systems for management of diseases including pelvic inflammatory disease, ectopic pregnancy and infertility [2,4,5].

In Australia, routine chlamydia testing using culture or direct immunofluorescence assays that detect elementary bodies by fluorescent microscopy [6,7] started in the 1980s. These tests were superseded by antigen detection using enzyme immunoassay [6,8-12] and in the 1990’s nucleic acid testing (NAT) [13-16] was introduced. Due to the superior analytical sensitivity of NAT over other technologies, it is considered the gold standard test for *C. trachomatis*[8] and is used universally in Australia for routine testing.

Pathology testing in Australia is covered by the national universal health insurance “Medicare”. Only Australian clinicians and some accredited healthcare professionals may request pathology testing. Several recommendations exist for opportunistic and routine chlamydia screening in priority populations in various clinical settings; whilst guidelines differ, the populations most commonly highlighted for routine and/or opportunistic screening include sexually active young people, Indigenous people, recent sexual contacts of infected men and women and gay, bisexual and other men who have sex with men (MSM) [1-4]. For chlamydia screening in men, a first-void urine, taken more than one hour after previous void, or urethral swab, but only when a discharge is present, are recommended [17]. Endocervical or vaginal swabs or first-void urine samples are recommended for women. Ano-rectal swabs are recommended if clinically indicated, particular with MSM. In addition, MSM are recommended to have annual rectal swab irrespective of symptoms or more frequently if engaging in unprotected anal sex or if more than 10 partners in a year [17,18]. Pharyngeal swabs for chlamydia are not generally indicated. Parental consent is not required from young people aged <18 years when assessed by a clinician to be competent to give consent [19].

Despite passive surveillance of positive cases in Australia, until recently there was no nationally-coordinated systematic surveillance system to monitor the number of tests conducted or enhanced surveillance data to elucidate who was being tested. These are important epidemiological indicators that are not commonly collected nor reported but that are particularly useful for the interpretation of positive chlamydia tests [20]. The availability of denominator data is particularly useful because it allows the number of positive tests to be interpreted in the light of testing patterns, providing a positivity rate that can be used as an indicator of the long-term outcome of prevention programs. For example, without these denominator data, it is not possible to ascertain if the increase in chlamydia notifications over time is due to greater transmission [21,22], variation in bacterial strains [23,24] or laboratory technology [25,26], an increase in testing, or a mixture of each of these variables [20]. Prevalence studies could provide this information, but they are extremely costly to run. Furthermore, they are not able to provide an accurate picture of trends and practices of chlamydia testing in a population, an important measure in itself.

Understanding the epidemiology of chlamydia and identifying high-risk groups aids in the implementation of prevention and intervention programs. Priority populations in Australia include young people aged 25 years or less, men who have sex with men (MSM), Aboriginal and Torres Strait Island people, and sex workers [27]. Various health departments have implemented health promotion campaigns targeting priority populations [28-31] and have encouraged general practitioners to increase testing of young people [32-36]. Sentinel surveillance systems have generally found that the increase in chlamydia notifications was due to increases in both the number of tests and an increase in the proportion of positive test results [37-42]. However, these studies usually are confined to clinics managing individuals at higher risk of infection compare with the general population [37,43]. There is limited information on population-level changes in testing behaviour.

In 2007, the Australian Collaboration for Chlamydia Enhanced Sentinel Surveillance (ACCESS) program was established [44]. Described in detail elsewhere [44], ACCESS is referred to as being “enhanced” because it consists of five clinical sentinel surveillance networks: family planning clinics, Aboriginal community controlled health services, sexual health clinics, antenatal clinics, and general practice clinics; plus a laboratory network. The aim of the ACCESS Laboratory Network was to describe characteristics and trends in chlamydia testing and diagnosis in public and private laboratories nationally, providing stakeholders with a large quantity of population data on testing frequency and outcomes, and demographics of people who undergo chlamydia testing in most states of Australia. This paper presents the findings of the laboratory network during its pilot phase between 2008 and 2010.

## Methods

### Recruitment

Data were collected through the ACCESS Laboratory Network, a national network of public and private laboratories. Ethics approval was granted by the Alfred Hospital Ethics Committee (Project 90/12). As the data extracted contained no patient identifers, consent from each individual included in the study was not required. All Australian laboratories testing for chlamydia at the time of recruitment (approximately 40 laboratories) were invited to participate at relevant workshops, newsletters, and through email, telephone calls and on-site visits. Of the laboratories approached, 15 laboratories participated in the Laboratory Network, including five from Victoria, four from Queensland, three from New South Wales, two from South Australia and one from Tasmania. Four were private and 11 were public laboratories.

Participating laboratories employ a range of commercial and in-house NAT detecting both chlamydia and gonorrhoea, and usually both tests are performed irrespective of the request. All laboratories have participated in one or more external quality assessment scheme and a quality control program provided by NRL. During the period of time of the study, some laboratories used assays that were unable to detect some known variants of chlamydia, however there were very few imported cases circulating in the Australian population at that time [45].

### Data collection, encryption and transfer

Data collected from the 15 participating laboratories included all chlamydia test records conducted between 1 January 2008 and 31 December 2010. The laboratories that declined the invitation to participate did so due to lack of time or resources. Data were extracted in March 2011. Two laboratories did not provide data for 2010. Data were collected directly from laboratory information systems (LIS) using electronic data extraction software (GRHANITE; University of Melbourne, Faculty of Medicine, Australia) [46]. GRHANITE automatically accessed the extracted data file that was stored within the security of the LIS and encrypted the patient identifiers into a non-reversible and de-identified statistical linkage keys that are unique to each individual. Core variables extracted included the de-identified statistical linkage keys, gender, date of birth, postcode of residence, sample type, date of request, date of result, and test result. Tests results were provided on all patients irrespective of age by all participating laboratories except one Victorian laboratory where the data in 2008 only pertained to those individuals aged 16 to 29 years. The de-identified data were then electronically transmitted to a secure server located at the Burnet Institute, Melbourne, Australia, decrypted and uploaded to an MS SQL database.

### Data analysis

Data were excluded when patient’s age was greater than 90 years, 0 or left blank; if the result was equivocal or missing or if there was a site/gender mismatch e.g. a cervical swab from a male patient, although this may have excluded some valid results from transgender individuals. The anatomical sites were categorised into: cervical (swabs), cervical (thin prep), eye, genital (including all non-cervical, −urethral and -vaginal samples), rectal, throat, urethral, urine, vaginal, other (including skin, internal organ, body fluids) and unknown.

All test results, including repeat tests, were used in the analysis. The percentage positive for chlamydia was calculated by dividing the total number of positive results by the total number of tests performed, stratified by calendar year, age, sex and anatomical site. Analysis by anatomical site included all specimens, but in age and sex specific analysis when an individual had multiple sites sampled on the same day, only one test (episode) was counted. The testing episode was considered chlamydia positive if any of the specimens were positive.

Stata statistical analysis software (StataCorp, Tx., USA) was used for all analyses. Trends over time were determined using a chi-squared (Chi^2^) test for trends. Binomial 95% confidence intervals (CI 95) were calculated for these estimates.

## Results

### Testing data received

A total of 628,295 chlamydia tests were collected, of which 11,575 (1.8%) tests were excluded due to: patient’s age (>90 years or 0 years) or age was unavailable (n = 3,243), unassigned gender (n = 2,030), equivocal or missing results (n = 5,035) and site/gender mismatch (n = 1,267). Data from the remaining 616,720 tests were included in the analysis of results by specimen site. Of the thirteen laboratories that provided chlamydia test results for all three years there were 167,453 tests in 2008, 228,202 in 2009 and 198,948 in 2010. A further 24,094 specimens, which represented samples taken from multiple anatomical sites during the same testing episode, were excluded for analysis by age and sex, leaving a total of 592,626 tests.

### Number and type of tests

**Figure 1:**
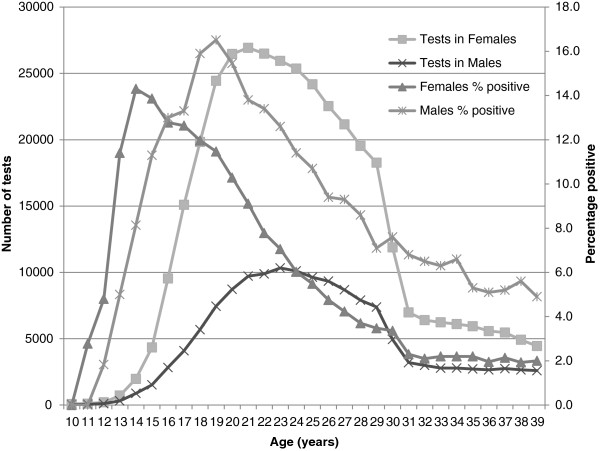
Chlamydia testing numbers and positivity rates by age and sex, 2008–2010.

**Figure 2:**
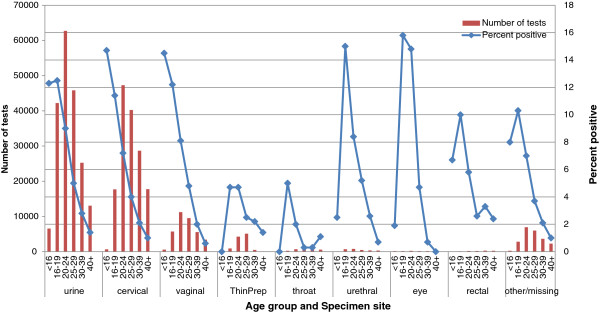
Chlamydia test data on female patients by anatomical site and age.

**Figure 3:**
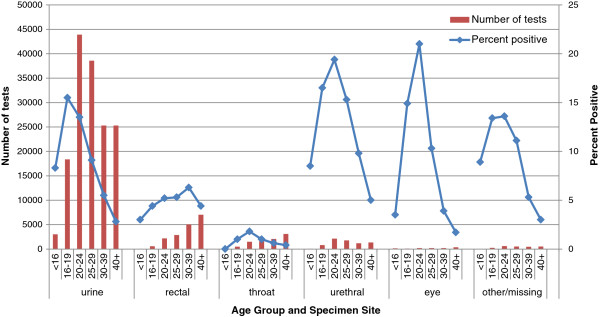
Chlamydia test data on male patients by anatomical site and age.

Of the 592,626 testing episodes, 415,069 (70%) tests were from female and 177,557 (30%) from male patients; 417,570 tests (70%) were from individuals aged 16–29 years (Table 1). The highest number of tests was among men and women aged between 20 and 29 (Figure 1). In female patients, 55.4% of samples were urine specimens and 29.3% were cervical (swabs) (Figure 2). The majority (79.6%) of samples collected from male patients were urine specimens (Figure 3).

**Table 1:** Chlamydia test data collected from 15 Australian laboratories obtained over a three-year period displayed by year

**Sex and age in years**		**Chlamydia tests**			**Positive tests**			**Positivity rates**			
		**2008**	**2009**	**2010**	**2008**	**2009**	**2010**	**2008**	**2009**	**2010**	P*
		**(n)**	**(n)**	**(n)**	**(n)**	**(n)**	**(n)**	**(%)**	**(%)**	**(%)**	
Female patients	<16	2,174	3,172	2,761	279	352	367	12.8	11.1	13.3	0.56
	16-19	21,403	25,265	22,190	2,539	2,937	2,826	11.9	11.6	12.7	**<0.01**
	20-24	40,124	48,076	42,943	3,237	3,780	3,562	8.1	7.9	8.3	0.2
	25-29	32,570	38,999	34,178	1,468	1,723	1,457	4.5	4.4	4.3	0.14
	30-39	16,161	27,834	20,009	382	643	554	2.4	2.3	2.8	**0.02**
	40+	8,711	16,734	11,765	93	218	127	1.1	1.3	1.1	0.98
	all	121,143	160,080	133,846	7,998	9,653	8,893	6.6	6	6.6	0.50
Male patients	<16	895	1,145	1,242	77	82	111	8.6	7.2	9	0.64
	16-19	6,019	7,307	6,710	856	1,072	1,102	14.2	14.7	16.4	**<0.01**
	20-24	14,789	17,794	16,205	1,979	2,328	2,165	13.4	13.1	13.4	0.99
	25-29	13,431	15,597	13,922	1,261	1,404	1,253	9.4	9	9	0.28
	30-39	8,070	12,458	9,583	488	775	581	6.1	6.2	6.1	0.91
	40+	8,425	13,747	10,218	287	513	314	3.4	3.7	3.1	0.17
	all	51,629	68,048	57,880	4,948	6,174	5,526	9.6	9.1	9.6	0.93
**Total**		172,772	228,128	191,726	12,946	15,827	14,419	7.5	6.9	7.5	0.56

*The number of tests per year and the percentage positivity by age group and gender are shown with the differences between the percentage positivity estimated using Chi-squared and expressed as *p* value and the significant values presented in bold type.

### Chlamydia positivity by age and sex

There were 43,192 (7.3%, 95 CI: 7.2 - 7.4%) chlamydia positive tests; 26,544 (6.4%, 95 CI: 6.3-6.5) in female and 16,648 (9.4% (95 CI: 9.2-9.5) in male patients. Between 2008 to 2010, the chlamydia positivity in female patients aged 16–29 years was 7.7% (95 CI: 7.6-7.8). The highest positivity rate among females was among 14 and 15 year olds (14.3% [95% CI 12.8-15.9] and 13.9% [95% CI 12.9-15.0] respectively) and positivity rate declined as the age of the patients increased (Figure 1). Chlamydia positivity in male patients aged 16–29 years over the three year period was 12.0% (95% CI: 11.8-12.2), peaking at 16.5% (95% CI 15.6-17.3) positive at age 19 years (Figure 1). From 2008 to 2010 there was a significant increase in chlamydia positivity for 16 to 19 year old females (11.9% to 12.7%, p < 0.01) and males (14.2% to 16.4%, p < 0.01) and female patients aged 30–39 years (2.4%-2.8%, *p* = 0.02).

### Positivity by specimen type and anatomical site

Urethral swabs, which are recommended only when a discharge is present, gave the highest positivity rate for all anatomical sites in both female 7.7% (Figure 2) and male patients 13.8% (Figure 3). The overall positivity rate for eye swabs was 6.3% (Figure 2) for female and 7.9% (Figure 3) for male patients.

In 16–29 year old female patients, the chlamydia positivity was 10.0% in urethral swabs, 8.8% in urine specimens, 7.8% in vaginal swabs, 6.7% in cervical swabs, 5.2% in rectal swabs, 3.6% for cervical (thin prep) and 1.7% in throat swabs over the three-year period. Male patients aged between 16–29 years had chlamydia positivity of 17.4% in urethral swabs, 12.2% in urine specimens, 5.2% in rectal swabs and 1.3% in throat swabs. The highest chlamydia positivity in male rectal swabs was seen in those aged 30–39 years (6.3%).

## Discussion

To our knowledge, the ACCESS Laboratory Network is the first national surveillance system designed to collect and analyse chlamydia testing data that are representative of a range of clinical sites, including general practice, antenatal clinics, sexual health clinics and family planning clinics as well as hospital in- and out-patients clinics. Data collected by the ACCESS Laboratory Network is unique in size and scope, providing data on almost 630,000 tests from 590,000 episodes from 15 Australian laboratories over a three-year period. In 2009, 62,660 chlamydia infectious were reported to state and territory health authorities under the provisions of the public health legislation in their jurisdiction [47]. In the same year, the ACCESS Laboratory Network collected 15,827 positive results, representing just over 25% of all chlamydia notifications nationally. The participating laboratories provided a good representation of chlamydia testing across Australia as the population serviced included high risk individuals, general practice, and hospital in- and out-patients. Although coverage was not complete, there is no other system in Australia which can provide such a large, representative overview of chlamydia testing and positivity.

Data presented here reveal higher and increasing chlamydia positivity rates among young people, particularly those aged younger than 16 years, and highlights the importance of ongoing population-level surveillance to monitor trends in testing and positivity in a broader population than traditionally captured in sentinel surveillance systems.

Of particular note, the highest positivity rate was found in female patients aged 14–15 years, although relatively few tests were performed. It is quite possible that only individuals in this demographic that have engaged in high risk activities are seeking testing; therefore increasing the positivity rate. However, there are limited data on chlamydia testing, positivity, and risk behaviours among 12–15 year olds as many studies are limited to collecting data on individuals aged 16 years and older. Evidence suggests that many young people aged younger than 15 years are sexually active - 23% of young people surveyed at a music festival reported their first sexual intercourse at 15 years or less (unpublished data) – and thus data on this age group collected in the ACCESS Laboratory Network is both unique and paramount to monitoring emerging trends in infection. The high positivity rate found in this study highlights the need for ongoing research with this vulnerable age group, as well as potential modifications to the target ages for chlamydia control programs. Even though these data are anonymised, ethics committees would not allow the collection of data on patients less than 16 years in the ACCESS clinical networks: this policy should be reconsidered.

Our findings reveal the majority of chlamydia tests were performed on female patients, though positivity was higher among male compared with female patients. This finding is consistent with studies that show that approximately 50% of men with chlamydia in clinical settings will have symptoms of urethritis, while asymptomatic men with chlamydia are more often tested after being a contact of a woman with chlamydia [48,49].

An important finding presented here is the significant increase in positivity rate from 2008 to 2010 in both female and male patients aged 16–19 years. This may be due to an increase in transmission in this age group related to changes in sexual behaviour, increased targeting of higher risk individuals within this population or a bias in the collection of data [21,42]. An increase in positivity was also observed among female patients aged 16–19 years in the ACCESS General Practice Network (8.7% in 2008 to 12.6% in 2010 among females) whilst positivity among male patients aged 16–19 years remained stable (19.6% in 2008 and 19.0% in 2010) [50]. The Victorian Network for Sentinel Surveillance of STI also found an increase in chlamydia positivity among women tested (from 5.1% to 6.3%) [42]. Continued monitoring is needed to ascertain if this increase in positivity rates continues over time and if an increase is also observed among young men in order to inform and evaluate national and state chlamydia control programs.

Australian chlamydia prevalence estimates vary by age group, population and setting where testing was conducted. A recent systematic review estimated the pooled prevalence for women <25 years in studies conducted post-2005 in community or general practice settings, was 5.0% [51]; our study suggests a higher positivity among young women however is likely larger due to the inclusion of tests conducted at sexual health clinics in the data, and therefore representative of prevalence at a range of clinical settings. Chlamydia positivity in women aged 16–29 years was higher in urine (8.8%) and vaginal swabs (7.8%) than cervical swabs (6.7%), despite chlamydia infecting the columnar epithelial cells of the cervix. The lower positivity rate in cervical swabs may be due to sampling of the cervix being performed at the time of cytological screening whereas urine and vaginal specimens were collected when the patient is symptomatic or perceived to be at high risk. This is supported by the low rates of positivity in cervical (thin prep) specimens (3.6%) and that the positivity rate of cervical swabs in women over the age of 30 years is less than 2%.

Almost 80% of samples from male patients tested for chlamydia were urine specimens. Over the three-year period chlamydia positivity was highest in men aged 16–19 years (15.1%) although men aged 20–24 years had more tests performed. Of concern, the positivity rate in men aged 16–19 years increased significantly over the three year period. An increase in positivity among young males was also observed in the ACCESS Family Planning Clinic Network [48,52] but not in the ACCESS General Practice Clinic Network [50]. The increased positivity could potentially be a result of improved targeting of chlamydia testing or due to a real increase in the population prevalence; targeted studies are required to investigate this further. Fewer males were tested than females, which is likely related to different health seeking behaviour, with men testing more in response to symptoms or contacts, and women presenting more routinely for opportunistic asymptomatic screening during health service visits for other reasons such as seeking contraceptive advice or cervical screening. The high positivity found among young men suggests that increased screening is warranted.

Rectal swabs from male patients (5.2% positivity) enabled the ACCESS Laboratory Network to assess a proxy measure of chlamydia positivity in MSM. Estimates of chlamydia positivity rates in MSM are limited in Australia, with ongoing estimates with large sample sizes lacking [53]. This finding was lower than estimates of positivity (at any anatomical site) among MSM from the ACCESS sexual health service network (7.2%) [44] and Victorian Primary Care Network for Sentinel Surveillance (6.4%) [54], both of which estimate prevalence for MSM that seek sexual health care at either sexual health services or gay men’s health clinics and are thus are not representative of the broader MSM population. The lower positivity rate may be due to the inclusion of lower-risk MSM in the study population compared with higher risk men that present to sexual health services and gay men’s clinics. Furthermore, including only rectal chlamydia is likely to underestimate chlamydia positivity among MSM by excluding urethral, pharyngeal, and other sites. Nonetheless, this finding highlights the potential for data collected through a laboratory network to complement sentinel surveillance data when interpreting trends in positivity in priority populations.

The highest positivity rate of male rectal swabs was in those aged 30–39 years and is consistent with Victorian data on chlamydia positivity by age [54]. However, the chlamydia positivity rates observed for rectal and throat swabs should be interpreted with caution [55-57].

The commercial chlamydia NAT assays used in laboratories are only validated for samples that have a high likelihood of containing infected epithelial cells e.g. cervical swabs, vaginal swabs or first stream urine specimens. However, the analysis showed that many other samples types, such as throat swabs and rectal swabs, are commonly referred to pathology laboratories. These sample types must be validated by the laboratory prior to being tested but often the validation may not occur or not done comprehensively due to the difficulties obtaining appropriate samples in sufficient quantity. That being said, a recent report indicates that rectal swabs are an effective sample type for the diagnosis of chlamydia infection [57].

In Australia, the only current general population data source for chlamydia testing is from Medicare Benefit Schedule data, which reported only 850,779 tests conducted in Australia between 2008 and 2010 (786,455 in the states and territories with ACCESS Laboratory Network sites); these data only include tests for which a Medicare benefit was claimed and exclude testing undertaken in state-funded public facilities as well as many sexual health services. It also provides no data on positivity rates [58]. Therefore, data collected in this study is unique in that it collates data from a broader range of sites and is more representative of overall population-based chlamydia testing and positivity patterns.

The current study has some limitations. Despite the ACCESS Laboratory Network providing a very large sample size, the data reflect only a subset of the total testing in Australia. The actual proportion is unable to be discerned due to the lack of data on chlamydia testing in Australia. One Victorian laboratory only reported results in 2008 for individuals aged 16–29 years due to installation issues and two laboratories were unable to provide data for 2010 due to changes in their LIS and in key personal. Due to the lack of continuity of data from these laboratories, only the rate of positivity was investigated in trends analysis. It is also acknowledged that the removal of site/gender mismatch may have excluded valid results from some transgender individuals. It is anticipated that future analyses of ACCESS Laboratory Network data will overcome this issue through the development of processes to identify and include individuals that identify as transgender.

Laboratories have only limited data on the patients that they are testing. In particular, data on whether patients belong to certain priority populations – Aboriginal and Torres Strait Islanders, sex workers, MSM (apart from the surrogate measure of rectal swabs on men) – are not available. That is why the ACCESS project complements these laboratory data with data from clinical networks. Further, all of these specimens are derived from patients who sought health care, either because of symptoms or perceived risk, so the data should be interpreted accordingly.

Despite these limitations, data presented here demonstrates the ACCESS Laboratory Network can provide unique and invaluable data. The GRHANITE system was implemented successfully in each of these laboratories and collated data that were meaningful and of high quality and large volume. The ACCESS Laboratory Network data set represents the most complete and extensive data set on chlamydia testing among the general population in Australia and will be an important resource for understanding chlamydia epidemiology into the future.

## Conclusion

The ACCESS Laboratory Network data are unique in both number and scope and are representative of chlamydia testing in both general practice and high-risk clinics. The use of GRHANITE allowed the extraction of testing and demographic data. Analysis of these data highlight much lower levels of testing in young people aged 20 years or less; in particular female patients aged less than 16 years, despite being the group with the highest positivity rate. Strategies are needed to increase the uptake of testing in this high-risk group.

## Competing interests

The authors declare that they have no competing interests.

## Authors’ contributions

WD organised and coordinated the participating laboratories, performed data analysis and was responsible for drafting the manuscript. ML performed statistical analysis and edited and contributed to the drafting of the manuscript. CvG was the responsible for the overall coordination of the project, made significant contributions to the drafting and editing of the manuscript. RG was involved in the initial design and implementation of the project, data analysis and manuscript editing. DB was the developer of the extraction software (GHRANITE) and responsible for the installation of software and data cleaning and storage processes. BD and MH conceived and designed the project, were responsible for overseeing the project and contributed to the editing of the manuscript, especially by providing clinical advice and references. All authors read and approved the final manuscript.

## Pre-publication history

The pre-publication history for this paper can be accessed here:

http://www.biomedcentral.com/1471-2334/14/325/prepub
